# PRAGMATIC IMPLEMENTATION OF AN ADVANCE CARE PLANNING INTERVENTION: FINDINGS FROM SHARING CHOICES

**DOI:** 10.1093/geroni/igad104.1525

**Published:** 2023-12-21

**Authors:** Daniel Scerpella, Jennifer Wolff, Joseph Gaugler

## Abstract

Advance care planning (ACP) is defined as a key task in ambulatory care for patients to share values, goals, and preferences for future medical care. Ideally ACP is revisited throughout the course of serious illness and engages family in discussions with clinicians. Most interventions, however, have not targeted primary care as a setting for ACP but have instead targeted a specific illness, conversation or setting such as the inpatient hospital or nursing home. Additionally, family is often not included in ACP discussions. This symposium will discuss critical components of the SHARING Choices cluster-randomized pragmatic trial, including the setup, monitoring, and analysis of this intervention within two primary care health systems located in the Baltimore-Washington DC metropolitan corridor. Each presenter will discuss key efforts conducted during the trial that highlight challenges, lessons learned, and novel approaches of embedding an evidence-based intervention within usual care settings targeting older adults 65 years of age and older. Preliminary analyses indicate that the ACP intervention was highly effective for increasing the likelihood of having advance directives in the patients’ electronic health records within 1-year of intervention initiation (odds ratio = 2.88, p < 0.001). Attendees of this symposium will take away relevant insights for implementing effective ACP and communication interventions within primary care settings as well as supporting outcome data. Our discussant will provide thoughts on the implications of these results for current primary care organizations and in the field of ACP and communication for older adults including those with dementia.

